# Variation at Genes Influencing Facial Morphology Are Not Associated with Developmental Imprecision in Human Faces

**DOI:** 10.1371/journal.pone.0099009

**Published:** 2014-06-10

**Authors:** Sonja Windhager, Helmut Schaschl, Katrin Schaefer, Philipp Mitteroecker, Susanne Huber, Bernard Wallner, Martin Fieder

**Affiliations:** 1 Department of Anthropology, University of Vienna, Vienna, Austria; 2 Konrad Lorenz Institute for Evolution and Cognition Research, Altenberg, Austria; 3 Department of Theoretical Biology, University of Vienna, Vienna, Austria; 4 Cognitive Science Platform at the University of Vienna, Vienna, Austria; 5 Department of Behavioural Biology, University of Vienna, Vienna, Austria; University of Utah, United States of America

## Abstract

Facial asymmetries are commonly used as a proxy for human developmental imprecision resulting from inbreeding, and thus reduced genetic heterozygosity. Several environmental factors influence human facial asymmetry (e.g., health care, parasites), but the generalizability of findings on genetic stressors has been limited in humans by sample characteristics (island populations, endogamy) and indirect genetic assessment (inference from pedigrees). In a sample of 3215 adult humans from the Rotterdam Study, we therefore studied the relationship of facial asymmetry, estimated from nine mid-facial landmarks, with genetic variation at 102 single nucleotide polymorphism (SNP) loci recently associated with facial shape variation. We further tested whether the degree of individual heterozygosity is negatively correlated with facial asymmetry. An ANOVA tree regression did not identify any SNP relating to either fluctuating asymmetry or total asymmetry. In a general linear model, only age and sex—but neither heterozygosity nor any SNP previously reported to covary with facial shape—was significantly related to total or fluctuating asymmetry of the midface. Our study does not corroborate the common assumption in evolutionary and behavioral biology that morphological asymmetries reflect heterozygosity. Our results, however, may be affected by a relatively small degree of inbreeding, a relatively stable environment, and an advanced age in the Rotterdam sample. Further large-scale genetic studies, including gene expression studies, are necessary to validate the genetic and developmental origin of morphological asymmetries.

## Introduction

### Aim of the study

In a recent study by Liu and co-workers, 102 single nucleotide polymorphism (SNP) loci were associated with variation in the mid-face (i.e., relative position and shape of cheekbones, nose and eyes) of humans with European ancestry [1]. Five of these SNPs reached genome-wide significance. These SNP loci are in close vicinity to genes that were reported to play a key role in human facial development. In the current study, we use the genetic and morphometric data published in Liu et al. [1] to analyze, for the first time, the association between SNP variation and developmental instability.

For bilaterally symmetric traits, developmental instability during non-pathological growth is commonly estimated by the degree of individual morphological asymmetry, computed here as the shape difference (Procrustes distance) between a facial configuration and its reflection [2]. Within a population, this total amount of asymmetry (TA) usually is decomposed into two components: directional asymmetry (DA; the average asymmetry pattern in the population) and fluctuating asymmetry (FA; individual deviations from the average asymmetry pattern). FA consists of small, random asymmetries generally assumed to reflect an organism's inability to cope with environmental and genetic perturbations during ontogenetic development. Heterozygosity at protein-coding loci or at markers-linked-to-functional loci (i.e., non-random association) is assumed to increase the ability to compensate for genetic and environmental stress. This enables a more stable development which, in turn, leads to a more symmetric adult phenotype (overdominance) [3].

We thus assessed the association of facial TA and FA with (i) variation at the 102 SNPs reported to covary with facial shape as well as with (ii) heterozygosity (i.e., gene diversity) estimated from these SNPs. Our sample consists of 3215 adult humans of both sexes from non-isolated, non-inbred human samples of the “Rotterdam Study” cohorts studied by Liu et al. [1].

### The candidate genes

Liu et al. determined the following candidate genes to be related to adult human mid-facial variation [1]: the PR domain containing 16 (*PRDM16*) gene, paired box 3 (*PAX3*), tumor protein p63 (*TP63*), collagen alpha-1 (XVII) chain (*COL17A1*), and the uncharacterized gene locus chromosome 5 open reading frame 50 (*C5orf50*). *PRMD16*, *PAX3* and *TP63* encode transcription factors, and *COL17A1* encodes the alpha chain of type XVII collagen, which is a transmembrane protein.

*PRDM16* had been previously linked to orofacial development in general, and cleft palate formation in particular (e.g., [4], [5] in mice). Common variants of *PAX3* have recently been related to relative *Nasion* position in humans [6], while rare variants were found to play a role in the Waardenberg syndrome, which is often accompanied by a broadening of the nasal root and an increased intercanthal distance [7], [8]. Furthermore, a missense mutation in the paired domain of *PAX3* results in the craniofacial-deafness-hand syndrome, characterized by small and short noses as well as absence or hypoplasia of the nasal bones [9], [10]. Also *TP63* is involved in orofacial clefts when morphogenesis is altered because of heterozygous mutations [11]. The genes *C5orf50* and *COL17A1* were related with facial characteristics for the first time in Liu et al. [1].

With respect to facial morphology, the *PRDM16*-corresponding SNP (rs4648379) was associated with the alae of the nose and their distance to the tip of the nose (*Alare*–*Pronasale*), the SNPs subsumed under *PAX3* (represented by rs974448) with the distances between *Nasion* and the centers of the eyeballs, and the SNP related to *TP63* (rs17447439) with the distance between the centers of the left and right eyeballs. The distances between *Zygion* and *Nasion* as well as the distances between the center of the eyeballs and *Nasion* were related to an SNP thought to represent *C5orf50* (rs6555969). Finally, the latter two distances were also detected in relation to an SNP categorized as corresponding to *COL17A1* (rs805722) [1].

### Developmental imprecision

Deviations from perfect symmetry in bilaterally symmetrical facial traits are not only caused by detrimental genetic variants and mutations on single locations, but may occur for various genetic and environmental reasons within the non-pathological range of variation. Total asymmetry (TA) in general, and facial fluctuating asymmetry (FA) in particular, are well-established indicators for this kind of developmental instability or imprecision ([12] for a review). While environmental stressors typically include pathogens and deficient nutritional supply (meta-analyses: in insects [13], in humans [14]), genetic conditions that have been associated with increased asymmetry in humans are homozygosity and endogamy (e.g., [15]–[18], but see [19]). Rather than midlife socioeconomic status, poorer socioeconomic status during childhood was significantly correlated with lower facial symmetry in adulthood [20]. This supports a developmental perspective. As the genetic evidence for effects on facial asymmetry in humans is mainly based on demographic data (relatedness, island populations), this study set out to use SNPs in a large cohort.

### Objectives and predictions

The first objectives of our study are to relate genetic variation at (i) the 102 SNP loci as well as at (ii) the five SNPs that reached genome-wide significance with regard to European facial shape variation (based on the data from [1]) to facial FA and TA. No *a priori* prediction about which allele might promote facial symmetry could be derived. For the second objective of this study, we hypothesized that the degree of individual heterozygosity, as assessed through the 102 SNP loci, should be negatively correlated with facial FA and TA.

## Results

The ANOVA tree regression identified no SNP (among the 102 SNPs) that were significantly associated with FA or TA. We therefore do not present any multivariate models based on all 102 SNPs here.

**Figure 1:**
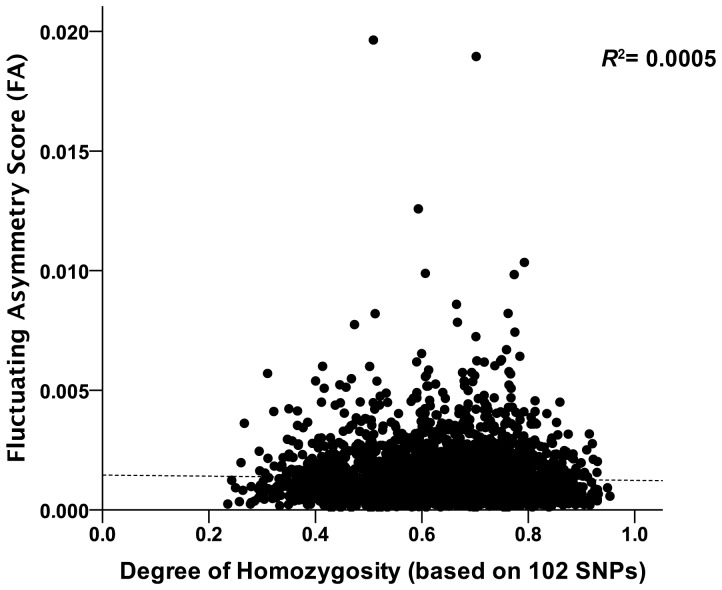
Lack of correlation between homozygosity by loci (HL) and Procrustes FA score (*N* = 3215). Individual homozygosity (based on 102 SNPs) is not correlated with facial FA inferred from the nine 3D facial landmarks previously used by Liu et al. [1]. The dashed line is the regression line (*r* = 0.024, *p* = 0.17, *N* = 3215). The same holds true for total asymmetry, because DA was very small, and thus TA and FA were strongly correlated (*r* = 0.98).

In the linear model including age, sex, homozygosity by loci (HL), cohort, as well as the 5 SNPs associated with mid-facial distances in the study of Liu et al. [1] with either FA or TA as the dependent variable, none of the SNPs reached genome-wide significance (*P*<10^−8^, Table 1). Neither FA nor TA were significantly associated with HL (see Figure 1 for FA). In line with the existing literature though, men are found to be more asymmetric than women, and both FA and TA scores increase with age (Table 1). In terms of cohorts, RS1 obtained the higher scores (*P* = 0.05 for FA and *P* = 0.04 for TA, respectively). All one-way interactions between sex, age and HL are non-significant and were therefore removed from the final model (Table 1).

**Table 1:** Regressions of mid-facial FA and TA on demographic and genotype data (*n* = 3215).

		Fluctuating Asymmetry (FA)				Total Asymmetry (TA)			
		Estimate	Std. Error	*t* value	*P*	Estimate	Std. Error	*t* value	*P*
(Intercept)		0.00078	0.00021	3.64300	0.00027	0.00074	0.00021	3.47000	0.00053
sex male	(ref. female)	0.00022	0.00004	5.26600	0.00000	0.00023	0.00004	5.52700	0.00000
age		0.00001	0.00000	3.01800	0.00256	0.00001	0.00000	3.37300	0.00075
HL		−0.00007	0.00018	−0.40100	0.68817	−0.00005	0.00018	−0.25800	0.79629
cohort RS2	(ref. RS1)	−0.00010	0.00005	−1.93400	0.05319	−0.00010	0.00005	−2.01100	0.04439
rs4648379	1 (ref. 0)	0.00009	0.00005	1.79200	0.07321	0.00008	0.00005	1.62000	0.10526
rs4648379	2 (ref. 0)	−0.00006	0.00008	−0.71000	0.47773	−0.00006	0.00008	−0.81200	0.41684
rs974448	1 (ref. 0)	−0.00003	0.00005	−0.69400	0.48771	−0.00005	0.00005	−0.95900	0.33788
rs974448	2 (ref. 0)	−0.00006	0.00013	−0.46600	0.64151	−0.00009	0.00013	−0.66500	0.50594
rs17447439	1 (ref. 0)	0.00001	0.00008	0.15100	0.87987	0.00002	0.00008	0.20000	0.84166
rs17447439	2 (ref. 0)	0.00095	0.00068	1.40600	0.15976	0.00085	0.00068	1.26300	0.20657
rs6555969	1 (ref. 0)	−0.00001	0.00004	−0.33100	0.74088	−0.00002	0.00004	−0.39100	0.69566
rs6555969	2 (ref. 0)	−0.00001	0.00007	−0.08900	0.92912	0.00000	0.00007	0.00500	0.99576
rs805722	1 (ref. 0)	0.00007	0.00005	1.41300	0.15774	0.00008	0.00005	1.59700	0.11026
rs805722	2 (ref. 0)	0.00010	0.00011	0.88100	0.37852	0.00009	0.00011	0.83500	0.40368

FA and TA were each regressed on the 5 SNPs (rs4648379, rs974448, rs17447439, rs6555969, rs805722), sex, age, HL and cohort. Genotypes are encoded accordingly to Liu et al. [1]: 0 = AA, 1 = AB and 2 = BB (their suppl. table 6).

### Homozygosity compared to 1000 Genomes samples

The average HL for the sample used by Liu et al. [1] resembles the averages for the European populations included in the 1000 Genomes project. Also as expected, individuals with “*African south of the Sahara ancestry*” have, on average, lower HL scores than individuals from “*East Asia*” (Table 2). The population variation in HL scores (percentiles) is comparable between the samples used by Liu et al. [1] and the 1000 Genomes samples (Table 3), assuring that the sample used by Liu et al. is not skewed towards lower or higher heterozygosity.

**Table 2:** Sample mean and variation of homozygosity by loci (HL) of the Liu et al. data [1] compared to several other human populations from the 1000 Genomes project.

Population	AVG HL	Std. Error	*N*
Yoruba in Ibadan, Nigeria (YRI)	0.587	0.012	88
African Ancestry in Southwest US (ASW)	0.592	0.018	61
Luhya in Webuye, Kenya (LWK)	0.598	0.011	97
Puerto Rican in Puerto Rico (PUR)	0.616	0.021	55
***Toscani in Italia (TSI)***	***0.633***	***0.013***	***98***
***Finnish from Finland (FIN)***	***0.636***	***0.015***	***93***
***Utah residents with Northern and Western European ancestry (CEU)***	***0.642***	***0.013***	***87***
***"Liu et al. sample" RS1***	***0.643***	***0.002***	***2470***
***"Liu et al. sample" RS2***	***0.643***	***0.004***	***745***
***British from England and Scotland (GBR)***	***0.652***	***0.014***	***88***
Colombian in Medellin, Colombia (CLM)	0.658	0.016	60
Mexican Ancestry in Los Angeles, CA (MXL)	0.671	0.016	66
***Iberian populations in Spain (IBS)***	***0.680***	***0.033***	***14***
Japanese in Tokyo, Japan (JPT)	0.693	0.011	89
Han Chinese in Bejing, China (CHB)	0.696	0.011	97
Han Chinese South (CHS)	0.707	0.011	100

Population names, average HL, standard error of the mean, and the sample size are given. Bold italicized values indicate European populations and populations of European descent.

**Table 3:** Distribution of HL for the RS1 and RS2 samples used by Liu et al. [1] and the 1000 Genomes populations.

	Liu et al. [1]		
HL Percentiles	RS1 sample	RS2 sample	1000 Genomes
10	0.462	0.463	0.465
20	0.527	0.540	0.531
30	0.579	0.579	0.583
40	0.621	0.622	0.626
50	0.656	0.652	0.660
60	0.688	0.685	0.693
70	0.722	0.722	0.730
80	0.759	0.762	0.764
90	0.803	0.804	0.805

## Discussion

We found TA and FA to be significantly related to sex and age of the human individuals, but not to the genetic variants at the five SNPs linked to several genes that reportedly play a key role in mid-facial development. Also the degree of HL (assessed at 102 SNP loci) was uncorrelated with either TA or FA in the faces of these 3215 Rotterdam Study individuals. This absence of correlation cannot result from any bottom or ceiling effect of the Netherland samples because the sample averages and variances are well within the range of several other human populations of European ancestry from the 1000 Genomes project (Tables 2 and 3).

### The 5 SNP loci involved in mid-facial development

In the GWAS by Liu et al. [1], five SNP loci, which reached genome-wide significance, were found to be associated with distances in the mid-face. One of the five key SNPs under investigation, rs974448 at 2q36.1, is located about 60 kbp downstream of the *PAX3* gene. This SNP is situated in an AT-rich low-complexity sequence, but without known regulative function. However, this SNP is apparently in the same linkage disequilibrium (LD) block as the *PAX3* intronic SNP rs7559271, which was significantly associated with the *Nasion*-to-Midendocanthion distance (n-men 3D dist) in another study [6]. Thus, future research should analyze the functional variation of cis-regulatory elements of the *PAX3* gene (e.g., gene expression studies). Along this line, Attanasio and colleagues [21] showed some of the complex morphogenetic mechanisms in craniofacial development resulting from transcriptional enhancer sequence variation in murine experiments. The authors concluded that this kind of variation should also contribute to human facial shape variation. Two regions in *PAX3* presumably comprise sequence motifs for enhancer binding, but none of these sequences include any SNPs used in Liu et al. [1], or at least none of them have been made publicly available. Thus, we lacked genetic data for these potentially regulative acting sequences as well as experimental evidence that these motifs actually affect the *PAX3* gene expression. Furthermore, the other four studied SNPs have not been linked to specific processes or functions. Accordingly, the test for a correlation between variation at these loci and facial asymmetry was explorative and proved to be non-significant. Nevertheless, this analysis opens a wide array of prospects for future research that are discussed at the end of the article. Apart from these five SNPs, a promising candidate might be also the SNP rs10843104 at 12p11.22: it is linked to the parathyroid hormone-like hormone (PTHLH) gene, which is involved in regulating cellular and organ growth as well as in endochondral bone development, and is required for skeletal homeostasis.

### Genetic diversity

Most previous articles on genetic aspects of facial and dental asymmetry are based on the comparison of endogamous (and often isolated and inbred) populations with more exogamous ones (such as [16]–[18]). Typically, the degree of heterozygosity increases with the level of exogamy and outbreeding (see also [22]), which, in turn, is associated with lower levels of organismal asymmetry. This reflects an increased ability to buffer against genetic and environmental stressors.

The higher the degree of heterozygosity at protein-coding genes or at linked loci (non-random association), the better adapted the individual is to buffer against perturbations. In this context, three hypothetical mechanisms are suggested: i) “The direct effect hypothesis” states that multilocus fitness-enhancing heterozygosity may result from a functional overdominance at the locus *per se* [23], [24]. In the case of allozymes, this might occur when heterozygotes possess enzymes with different catalytic properties and thus are more biochemically efficient than homozygotes [25]. ii) “The local effect hypothesis” proposes a heterozygosity—fitness correlation according to associative overdominance. In this case, there is a genetic association (linkage disequilibrium) between a neutral marker and a marker under selection [23], [26]. And iii) “the general effect hypothesis” states that heterozygote advantage at the markers under study stems from costs of homozygosity at fitness loci distributed over the whole genome. The prerequisite is that marker and fitness loci are in identity disequilibrium, which is caused by variance in the inbreeding coefficient of individuals in the same population. Inbred individuals will be relatively homozygous throughout their genome because of recent allelic co-ancestry, and as such will also be homozygous at marker loci, whereas in relatively outbred individuals the coupling of heterozygosity at marker and fitness loci will be weaker (cf. [3], [27]).

With regard to our study, we did not find any known allozymatic association with the 102 SNPs related to facial morphology (source: NCBI, SNP data base; http://www.ncbi.nlm.nih.gov/projects/SNP/). Based on our data and the NCBI SNP database, we have also no hint for effects other than additive, because we did not find any known epistatic effects associated with the 102 SNPs discovered by the study of Liu et al. [1]. To conclude, we think that our available data (with the emphasis on available) do not quite fit in any of these three models, because they assume heterozygosity–fitness correlation, which we cannot identify. Furthermore, Chapman et al. demonstrated that even if heterozygosity-fitness correlations exist, they are very small (usually explaining less than 1% of the phenotypic variance) [3].

In an extremely rare 2×2 design, Schaefer et al. showed that both inbreeding and detrimental environmental conditions added to the observed asymmetry level using four subgroups [16]: the endogamous samples were more asymmetric than an exogamous sample from the same island, which in turn was more asymmetric than the exogamous mainland population (that had better access to medical care, etc.). Possible reasons why we found no association between the degree of heterozygosity and facial asymmetries in our study include that the Rotterdam samples lack substantial degrees of inbreeding. As a result, the observed degree of heterozygosity might be—on average—not low enough to significantly impact developmental pathways. It is also reasonable to assume that the environment of this Rotterdam population does not fluctuate enough for homozygosity to be disadvantageous. A third rationale concerns the relative impact of genetic effects on facial asymmetries, compared to the environmental stressors that accumulate over time. In line with our results, Otremski et al. and Penke et al. found elevated fluctuating asymmetry scores in elderlies [28], [29] (as opposed to a relatively consistent decrease of FA from birth to mature adulthood, [28]). Intuitively reasonable, yet lacking solid empirical evidence, it has been suggested, that different sides of the face can age differently [30], for example due to asymmetric sun exposure ([31] showing the extreme example of a truck driver's face). The effect of ageing on asymmetry also differs between facial features: the lateral asymmetry of the nose increases sharper and steadier with age than the one of the chin [32]. The lack of a significant main effect of HL in our models, however, shows that we could not detect an effect of HL on facial asymmetry, even if age was held constant. Nevertheless, additional individual life-history data will be necessary to further investigate this issue and possible interactions between genetic and environmental components. Clearly, the explanations listed above are not mutually exclusive.

### Limitations of the study and prospects for future research

The sample was initially not collected for investigating facial asymmetry, which potentially introduces some limitations of our study. The participants' age range (45 to 93 years) is far beyond early adulthood so that environmental effects with aging might have added to facial asymmetries (and potentially blurred genetic signals). The 102 SNPs were reported to be associated with the mid-facial landmarks used in the study, but the relatively small number of SNPs may limit the representative value in terms of an individual's total heterozygosity (cf. [33]). Also, the number of landmarks is relatively small and their intra- and interobserver reliability is unknown. Furthermore, the nose (associated with about half of the landmarks in this study) has recently been found to be subject to stabilizing selection in human populations [34]. Hence the effects of certain SNPs and of homozygosity on mid-facial asymmetry might be less pronounced than effects on the lower face and teeth, which were investigated in several key articles on developmental imprecision in endogamous and isolated populations. Follow-up studies should therefore consider younger subjects, a larger number of SNPs (including those that Fatemifar et al. recently associated with facial, eye, and nose widths as well as *Glabella*—Midendocanthion distance) [35], and loci of known functional relevance (e.g. immune genes). They should further pursue experimental work in mammal models such as mice. This might add to our understanding of genetic components in non-syndromic, normal-range developmental imprecision and their interaction with environmental stressors and disturbances. Another future puzzle is to explain the differences in asymmetry in genetically identical individuals brought up under highly similar conditions (e.g., [36]).

Despite all these limitations, the data provided by the study of Liu et al. [1] is the largest to-date available dataset for a non-isolated, non-endogamous human population that included both genetic markers and three-dimensional facial shape information. The association of SNPs to facial morphology on the one hand, and to candidate genes associated with orofacial cleft birth defects and mid-facial development on the other hand, made these SNPs valuable sources to study genetic aspects of developmental imprecision. Future research along these lines, however, will be necessary before genetic aspects such as single nucleotide polymorphisms and heterozygosity can be confirmed or ruled out in shaping human facial asymmetries.

## Material and Methods

### Participants

We used the data set provided by Liu et al. [1], including the three-dimensional coordinates of nine mid-facial landmarks as well as the 102 SNP genotypes of 3215 adult individuals of both sexes (aged from 45 to 92 years; mean = 59.5 years, SD = 8.0 years) from two cohorts of the “Rotterdam Study” (RS1 and RS2 sample, for a detailed sample description see [1]).

The Rotterdam Study is an ongoing prospective cohort study, which started in 1990, in order to follow mid-adults from the city of Rotterdam (the Netherlands) over time for a variety of diseases [37]. The study was approved by the Medical Ethics Committee of the Erasmus MC, University Medical Center Rotterdam. All participants gave their written informed consent. Altogether, data from 15,000 subjects was collected with GWAS data for 80% of them. A subset of participants were scanned on a 1.5 T General Electric MRI unit (GE Healthcare, Milwaukee, WI, USA), using 192 slices, a resolution of 0.49×0.49×0.8 mm^3^ (up sampled from 0.66×0.7×0.8 mm^3^ using zero padding in the frequency domain), a repetition time (TR) of 13.8 ms, an echo time (TE) of 2.8 ms, an inversion time (TI) of 400 ms, and a flip angle of 20°. The corresponding imaging protocol included a 3D T1-weighted fast RF gradient recalled acquisition in steady state with an inversion recovery prepulse [1], [38]. DNA samples were collected and purified as described in Kayser et al. [39]. Liu et al. then used a Principal components analysis of SNP micro-array data to identify ancestry outliers, which subsequently were removed [1]. The final sample is of exclusively northern/western European origin and included 3,215 RS participants who had both SNP microarray data and 3D MRI. The split into two cohort (RS1 and RS2) results from two waves of scanning and genotyping.

### Genetic diversity

Individual heterozygosity was estimated by the homozygosity by loci (HL) index [40] using R (library Rhh). This method weighs the contribution of loci depending on their allelic variability and is calculated as follows:
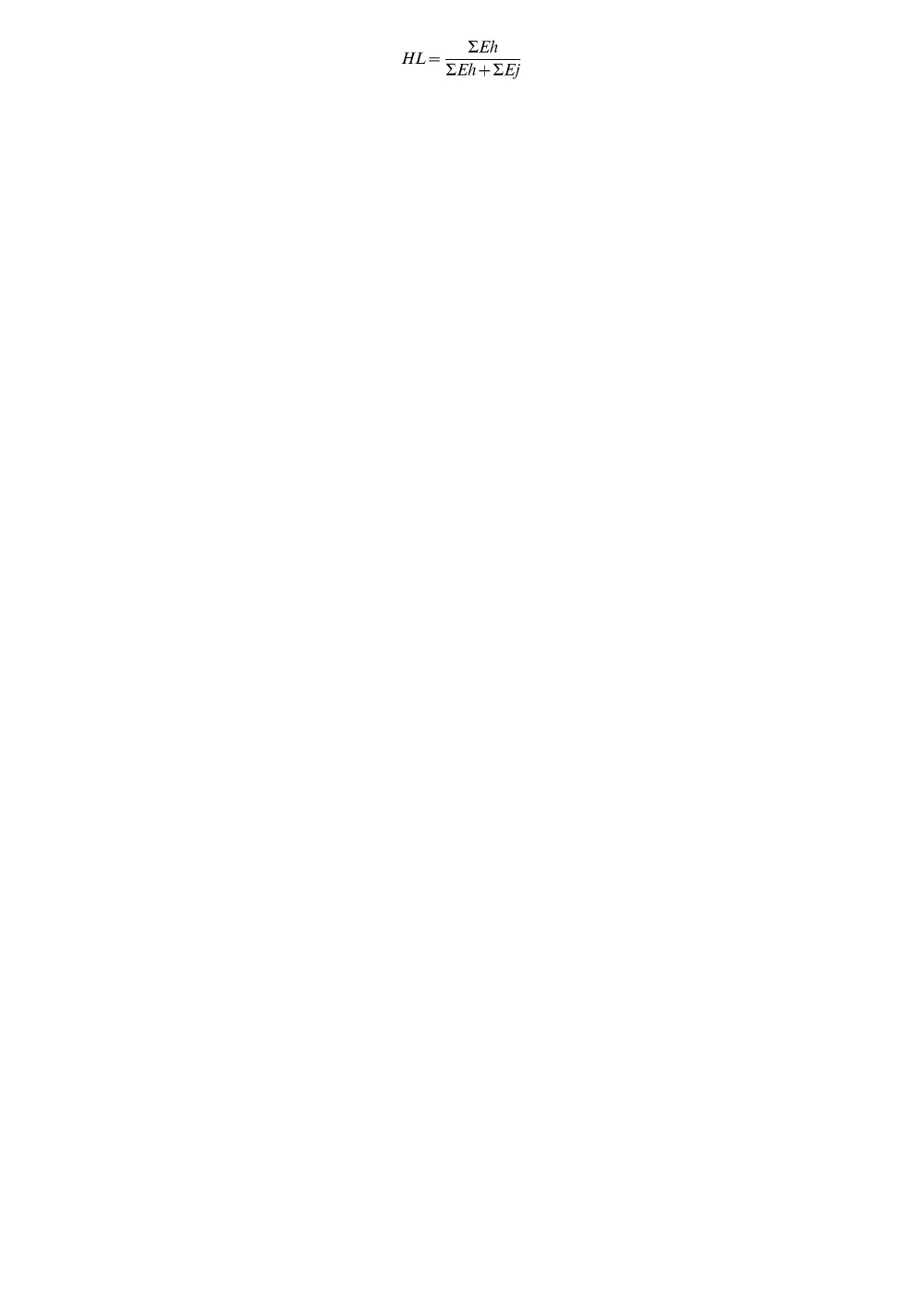
where *Eh* and *Ej* are the expected heterozygosities of the loci that an individual bears in homozygosis (h) and in heterozygosis (j), respectively. This index thus varies between 0 (all loci heterozygous) and 1 (all loci homozygous).

We compared the average and the variation of the HL scores of the RS1 and RS2 samples [1] with those of 14 other human populations in order to rule out bottom or ceiling effects. For this, HL was calculated based on the 102 SNPs for the following 14 human populations sampled by the 1000 Genomes project Phase I database (http://www.1000genomes.org/): African ancestry in Southwest USA (ASW, *n* = 61), Utah residents with Northern and Western European ancestry from the CEPH collection (CEU, *n* = 87), Han Chinese in Beijing, China (CHB, *n* = 97), Han Chinese South (CHS, *n* = 100), Colombian in Medellín, Colombia (CLM, *n* = 60), Finnish in Finland (FIN, *n* = 93), British in England and Scotland (GBR, *n* = 88), Iberian populations in Spain (IBS, *n* = 14), Japanese in Tokyo, Japan (JPT, *n* = 89), Luhya in Webuye, Kenya (LWK, *n* = 97), Mexican ancestry in Los Angeles, California (MEX, *n* = 66), Puerto Rican in Puerto Rico (PUR, *n* = 55), Toscans in Italy (TSI, *n* = 98), and Yoruba in Ibadan, Nigeria (YRI, *n* = 88). We extracted the SNP data from the 1000 Genomes database using SPSmart (http://spsmart.cesga.es/engines.php), which is a web-based tool for accessing and combining large-scale genomic databases of SNPs [41].

### Facial asymmetry

The positions of the nine mid-facial landmarks (*Zygion* left/right, *Alare* left/right, centers of the eyeballs, *Nasion*, *Pronasale*, *Subnasale*) were automatically localized on each scan ([42] for methodological details). Liu et al. report high test-retest correlations for this algorithm based on 40 subjects from another sample (QTIMS, Queensland Twin Imaging Study), who were scanned twice, (*r*>0.99) [1]. Although this analysis was not provided for the other samples including RS1 and RS2, there is no reason to question precision and repeatability in their case.

Based on the landmark coordinates, fluctuating asymmetry (FA) and total asymmetry (TA) were approached through a geometric morphometric method (e.g., [43], [44]). This way, analyses include several traits at the same time, and prevent the confounding of directional asymmetry with fluctuating asymmetry that has often afflicted past studies based on single linear measurements.

### Statistical analysis

FA and TA scores were each regressed on the genotype data of the 102 SNPs using an ANOVA tree regression (R library rpart). We further calculated two separate general linear models regressing i) the FA scores and ii) the TA scores on age, sex (encoded as male and female), homozygosity by loci (HL calculated on the basis of the 102 SNPs using the R library Rhh) and the 5 SNPs known to significantly influence distances within a face (rs4648379, rs974448, rs17447439, rs6555969, and rs805722; encoded as genotype, [1]). Hereby, we estimated all possible one-way interactions between sex, age and HL, and removed non-significant interactions stepwise from the model.

We used the free statistical package R (Version 3.01) for regressions and general linear models (GLMs), as well as Mathematica 8 to compute total and fluctuating asymmetry scores for each individual. According to Liu et al., genome-wide significance was set at *P*≤10^−8^ [1].

## References

[1] Liu F, van der Lijn F, Schurmann C, Zhu G, Chakravarty MM, et al (2012) A genome-wide association study identifies five loci influencing facial morphology in Europeans. PLoS Genet 8: e1002932. 23028347 10.1371/journal.pgen.1002932 PMC3441666

[2] Mitteroecker P, Gunz P (2009) Advances in Geometric Morphometrics. Evol Biol 36: 235–247.

[3] Chapman JR, Nakagawa S, Coltman DW, Slate J, Sheldon BC (2009) A quantitative review of heterozygosity-fitness correlations in animal populations. Mol Ecol 18: 2746–2765. 19500255 10.1111/j.1365-294X.2009.04247.x

[4] Bjork BC, Turbe-Doan A, Prysak M, Herron BJ, Beier DR (2009) Prdm16 is required for normal palatogenesis in mice. Hum Mol Genet 19: 774–789. 20007998 10.1093/hmg/ddp543 PMC2816611

[5] Warner DR, Horn KH, Mudd L, Webb CL, Greene RM, et al (2007) PRDM16/MEL1: A novel Smad binding protein expressed in murine embryonic orofacial tissue. Biochim Biophys Acta BBA – Mol Cell Res 1773: 814–820. 10.1016/j.bbamcr.2007.03.016 17467076

[6] Paternoster L, Zhurov AI, Toma AM, Kemp JP, St. Pourcain B, et al (2012) Genome-wide association study of three-dimensional facial morphology identifies a variant in PAX3 associated with Nasion position. Am J Hum Genet 90: 478–485. 22341974 10.1016/j.ajhg.2011.12.021 PMC3309180

[7] Pingault V, Ente D, Dastot-Le Moal F, Goossens M, Marlin S, et al (2010) Review and update of mutations causing Waardenburg syndrome. Hum Mutat 31: 391–406. 20127975 10.1002/humu.21211

[8] Read AP, Newton VE (1997) Waardenburg syndrome. J Med Genet 34: 656–665. 9279758 10.1136/jmg.34.8.656 PMC1051028

[9] Asher JH Jr, Sommer A, Morell R, Friedman TB (1996) Missense mutation in the paired domain of PAX3 causes craniofacial-deafness-hand syndrome. Hum Mutat 7: 30–35. 8664898 10.1002/(SICI)1098-1004(1996)7:1<30::AID-HUMU4>3.0.CO;2-T

[10] Sommer A, Bartholomew DW (2003) Craniofacial-deafness-hand syndrome revisited. Am J Med Genet A 123A: 91–94. 14556253 10.1002/ajmg.a.20501

[11] Rinne T, Brunner HG, van Bokhoven H (2007) p63-Associated Disorders. Cell Cycle 6: 262–268. 17224651 10.4161/cc.6.3.3796

[12] Polak M, editor (2003) Developmental instability: causes and consequences. Oxford, New York: Oxford University Press. 459 p.

[13] Beasley DAE, Bonisoli-Alquati A, Mousseau TA (2013) The use of fluctuating asymmetry as a measure of environmentally induced developmental instability: A meta-analysis. Ecol Indic 30: 218–226.

[14] Van Dongen S, Gangestad SW (2011) Human fluctuating asymmetry in relation to health and quality: a meta-analysis. Evol Hum Behav 32: 380–398.

[15] Livshits G, Kobyliansky E (1991) Fluctuating asymmetry as a possible measure of developmental homeostasis in humans: a review. Hum Biol 63: 441–466. 1889795

[16] Schaefer K, Lauc T, Mitteroecker P, Gunz P, Bookstein FL (2006) Dental arch asymmetry in an isolated adriatic community. Am J Phys Anthropol 129: 132–142. 16229029 10.1002/ajpa.20224

[17] Hershkovitz I, Ring B, Kobyliansky E (1992) Craniofacial asymmetry in Bedouin adults. Am J Hum Biol 4: 83–92. 28524406 10.1002/ajhb.1310040111

[18] Özener B (2010) Effect of inbreeding depression on growth and fluctuating asymmetry in Turkish young males. Am J Hum Biol 22: 557–562. 20309882 10.1002/ajhb.21046

[19] Livshits G, Smouse PE (1994) Relationship between fluctuating asymmetry, morphological modality and heterozygosity in an elderly Israeli population. In: Markow TA, editor. Developmental Instability: Its Origins and Evolutionary Implications. Dordrecht: Kluwer Academic Publishers. pp. 157–168.

[20] Hope D, Bates T, Penke L, Gow AJ, Starr JM, et al (2013) Symmetry of the face in old age reflects childhood social status. Econ Hum Biol 11: 236–244. 21820367 10.1016/j.ehb.2011.06.006

[21] Attanasio C, Nord AS, Zhu Y, Blow MJ, Li Z, et al (2013) Fine Tuning of Craniofacial Morphology by Distant-Acting Enhancers. Science 342: 1241006. 24159046 10.1126/science.1241006 PMC3991470

[22] Campbell H, Carothers AD, Rudan I, Hayward C, Biloglav Z, et al (2007) Effects of genome-wide heterozygosity on a range of biomedically relevant human quantitative traits. Hum Mol Genet 16: 233–241. 17220173 10.1093/hmg/ddl473

[23] Hansson B, Westerberg L (2002) On the correlation between heterozygosity and fitness in natural populations. Mol Ecol 11: 2467–2474. 12453232 10.1046/j.1365-294x.2002.01644.x

[24] Frydenberg O (1963) Population studies of a lethal mutant in *Drosophila Melanogaster* . Hereditas 50: 89–116.

[25] Mitton JB (1997) Selection in natural populations. Oxford, New York: Oxford University Press. 256 p.

[26] Coulson TN, Pemberton JM, Albon SD, Beaumont M, Marshall TC, et al (1998) Microsatellites reveal heterosis in red deer. Proc R Soc Lond B Biol Sci 265: 489–495. 10.1098/rspb.1998.0321 PMC1688908 9569667

[27] Miller JM, Coltman DW (2014) Assessment of identity disequilibrium and its relation to empirical heterozygosity fitness correlations: a meta-analysis. Mol Ecol 23: 1899–1909. 24581039 10.1111/mec.12707

[28] Otremski I, Katz M, Livshits G, Cohen Z (1993) Biology of aging in an Israeli population. 1. Review of literature and morphological variation analysis. Anthropol Anz 51: 233–249. 8215260

[29] Penke L, Bates TC, Gow AJ, Pattie A, Starr JM, et al (2009) Symmetric faces are a sign of successful cognitive aging. Evol Hum Behav 30: 429–437.

[30] Coleman SR, Grover R (2006) The anatomy of the aging face: volume loss and changes in 3-dimensional topography. Aesthetic Surg J 26: S4–9. 10.1016/j.asj.2005.09.012 19338976

[31] Gordon JRS, Brieva JC (2012) Unilateral dermatoheliosis. N Engl J Med 366: e25. 22512500 10.1056/NEJMicm1104059

[32] Sutton PR (1968) Lateral facial asymmetry-methods of assessment. Angle Orthod 38: 82–92. 5236055 10.1043/0003-3219(1968)038<0082:LFAMOA>2.0.CO;2

[33] DeWoody YD, DeWoody JA (2005) On the estimation of genome-wide heterozygosity using molecular markers. J Hered 96: 85–88. 15618305 10.1093/jhered/esi017

[34] Bookstein FL, Mitteroecker P (in press) Comparing covariance matrices by relative eigenanalysis, with applications to organismal biology. Evol Biol: 1–15.

[35] Fatemifar G, Hoggart CJ, Paternoster L, Kemp JP, Prokopenko I, et al (2013) Genome-wide association study of primary tooth eruption identifies pleiotropic loci associated with height and craniofacial distances. Hum Mol Genet 22: 3807–3817. 23704328 10.1093/hmg/ddt231 PMC3749866

[36] Vogt G, Huber M, Thiemann M, van den Boogaart G, Schmitz OJ, et al (2008) Production of different phenotypes from the same genotype in the same environment by developmental variation. J Exp Biol 211: 510–523. 18245627 10.1242/jeb.008755

[37] Hofman A, Murad SD, Duijn CMvan, Franco OH, Goedegebure A, et al (2013) The Rotterdam Study: 2014 objectives and design update. Eur J Epidemiol 28: 889–926. 24258680 10.1007/s10654-013-9866-z

[38] Ikram MA, van der Lugt A, Niessen WJ, Krestin GP, Koudstaal PJ, et al (2011) The Rotterdam Scan Study: design and update up to 2012. Eur J Epidemiol 26: 811–824. 22002080 10.1007/s10654-011-9624-z PMC3218266

[39] Kayser M, Liu F, Janssens ACJW, Rivadeneira F, Lao O, et al (2008) Three genome-wide association studies and a linkage analysis identify HERC2 as a human iris color gene. Am J Hum Genet 82: 411–423. 18252221 10.1016/j.ajhg.2007.10.003 PMC2427174

[40] Aparicio JM, Ortego J, Cordero PJ (2006) What should we weigh to estimate heterozygosity, alleles or loci? Mol Ecol 15: 4659–4665. 17107491 10.1111/j.1365-294X.2006.03111.x

[41] Amigo J, Salas A, Phillips C, Carracedo A (2008) SPSmart: adapting population based SNP genotype databases for fast and comprehensive web access. BMC Bioinformatics 9: 428. 18847484 10.1186/1471-2105-9-428 PMC2576268

[42] Boehringer S, van der Lijn F, Liu F, Günther M, Sinigerova S, et al (2011) Genetic determination of human facial morphology: links between cleft-lips and normal variation. Eur J Hum Genet 19: 1192–1197. 21694738 10.1038/ejhg.2011.110 PMC3198142

[43] Klingenberg CP, McIntyre GS (1998) Geometric morphometrics of developmental instability: analyzing patterns of fluctuating asymmetry with Procrustes methods. Evolution 52: 1363–1375. 28565401 10.1111/j.1558-5646.1998.tb02018.x

[44] Mardia KV, Bookstein FL, Moreton IJ (2000) Statistical assessment of bilateral symmetry of shapes. Biometrika 87: 285–300.

[45] R Core Team (2013). R: A language and environment for statistical computing. R Foundation for Statistical Computing, Vienna, Austria. Available: http://www.R-project.org/.

[46] Genomes Project Consortium (2012) An integrated map of genetic variation from 1,092 human genomes. Nature 491: 56–65. 23128226 10.1038/nature11632 PMC3498066

